# Coexistence of double seropositivity for MPO antibody and anti-GBM antibody in ANCA-associated vasculitis concurrent with multiple myeloma: A case report

**DOI:** 10.1097/MD.0000000000039021

**Published:** 2024-07-26

**Authors:** Hyeonjeong Lee, Jaeseok Yang, Jinykung Kwon, Mihwa Heo, Yaerim Kim, Jin Hyuk Paek, Hyeongchan Shin, Misun Choe, Seungyeup Han, Kyubok Jin

**Affiliations:** a Division of Nephrology, Department of Internal Medicine, Keimyung University School of Medicine, Keimyung University Kidney Institute, Daegu, Republic of Korea; b Division of Hematology-Oncology, Department of Internal Medicine, Keimyung University School of Medicine, Daegu, Republic of Korea; c Department of Pathology, Keimyung University School of Medicine, Daegu, Republic of Korea.

**Keywords:** ANCA-associated vasculitis, anti-GBM antibody, case report, MPO antibody, multiple myeloma

## Abstract

**Rationale:**

Immune-mediated vasculitis with 2 or more autoantibodies, for example, anti-proteinase-3, combined with anti-myeloperoxidase (MPO) or anti-glomerular basement membrane (GBM) antibodies, is extremely unusual. Furthermore, the coexistence of autoimmune vasculitis and hematological malignancies is uncommon. Herein, we describe a case of double-seropositive anti-neutrophil cytoplasmic antibody (ANCA) vasculitis with multiple myeloma.

**Patient concerns:**

A 79-year-old Asian man presented with persistent leg edema and kidney dysfunction. His kidney function rapidly decreased, and serologic test results showed higher titers of the anti-MPO antibody (54.7 IU/mL) and anti-GBM antibodies (>200 IU/mL). Additionally, the clinical features showed the possibility of monoclonal gammopathy with anemia and hyperglobulinemia. We performed kidney and bone marrow biopsy. Serum protein electrophoresis and immunofixation revealed no significant differences, but the results of the bone marrow smear were compatible with those of myeloma with 15% plasmacytosis. However, kidney biopsy showed diffuse crescentic glomerulonephritis without deposition of the immune complex or kappa/lambda chain.

**Diagnoses and Interventions:**

Finally, the patient was diagnosed with double-seropositive ANCA-associated glomerulonephritis and multiple myeloma. Given the patient’s performance status, we initiated low-dose steroid pulse therapy, followed by conservative management.

**Outcomes:**

While the pulmonary lesions showed improvement, the kidney function did not regain its previous state, prompting the initiation of kidney replacement therapy by hemodialysis. There has been a decrease in the levels of anti-GBM and anti-MPO antibodies since the initial diagnosis.

**Lessons:**

This case elucidates the complex interplay between ANCA-associated glomerulonephritis and hematologic malignancy and emphasizes the need for a nuanced treatment strategy considering its multifaceted clinical presentation.

## 1. Introduction

Anti-neutrophil cytoplasmic antibody (ANCA)-associated vasculitis (AAV) represents a spectrum of disorders characterized by pronounced inflammation of blood vessels, endothelial injury, and consequent tissue damage.^[1]^ The involvement is typically represented by crescentic glomerulonephritis (GN), which is closely associated with a poor prognosis and is clinically characterized by rapidly progressive glomerulonephritis (RPGN). Crescentic GN is classified into 4 categories on basis of the presence and position of immune deposits; AAV is commonly recognized as the most prevalent type, classified as type 3, and indicative of pauci-immune glomerulonephritis.^[2]^ Another crescentic GN, categorized as type 1, is an anti-glomerular basement membrane (GBM) disease characterized by the existence of circulating and deposited antibodies targeting antigens found in the basement membrane and affecting the glomerular and pulmonary capillaries.

AAV and anti-GBM disease can be distinguished on the basis of the presence of an immune complex. Although their incidence is extremely low, they can also develop concurrently, resulting in their classification as a type 4 crescentic GN. The annual incidence of AAV is approximately 20 cases/million population, whereas that of anti-GBM disease is 1.64 cases/million people.^[3,4]^ Furthermore, a subset of patients with AAV, constituting 5% of the overall AAV cohort, demonstrates dual positivity for AAV and anti-GBM disease.^[5]^ Double-positive patients, exhibiting characteristics of both AAV and anti-GBM disease, share demographic traits reminiscent of AAV, including an older age distribution and an extended duration of symptoms before diagnosis. Concomitantly, they manifest features similar to those of anti-GBM disease, for example, severe kidney impairment and increased incidence of lung hemorrhage during presentation.^[5]^

Despite the lack of certainty regarding the exact mechanism, multiple studies have reported an association between AAV and the development of lymphoproliferative diseases, including lymphoma and myeloma.^[6]^ Although the reported case numbers are limited, the majority involve elderly patients with concomitant acute kidney injury and poor kidney outcomes. Notably, these patients exhibit positive myeloperoxidase (MPO) antibodies, and a common feature is the confirmation of multiple myeloma (MM) independent of kidney pathology.

Here, we describe a distinctive case independent of previously documented cases of overlapping diseases: a patient with crescentic GN exhibiting positivity for both anti-GBM antibodies and MPO antibodies, accompanied by concurrent MM.

## 2. Clinical presentation

The patient was a 79-year-old Korean man with had leg edema for 1 month. He was taking clopidogrel, telmisartan, and spironolactone for a history of underlying stroke and essential hypertension, and his baseline kidney function had not yet been established. He was referred to a tertiary hospital because of sustained leg edema accompanied by kidney dysfunction (serum creatinine level, 1.49 mg/dL) and proteinuria, hypoalbuminemia (serum albumin level, 2.5 g/dL).On the day of admission, his vital signs were as follows: blood pressure, 114/64 mm Hg; pulse rate, 91 per minutes; respiratory rate, 24 per minutes; and body temperature, 36.3°C. Both lung sounds were clear; however, significant leg pitting edema was observed on physical examination. Laboratory test results revealed the following: hemoglobin level, 6.8 g/dL; blood urea nitrogen level, 46 mg/dL; serum creatinine level, 3.21 mg/dL; serum protein level, 7.3 g/dL; serum albumin level, 2.3 g/dL; and serum calcium level, 7.5 mg/dL (corrected calcium level 8.9 mg/dL); and lactic dehydrogenase level, 376 IU/L. On urinalysis, albuminuria was 1+ with microscopic hematuria. The spot urine protein-creatinine ratio was 1.09 g/gCr, and 24-hour urine protein level was 1.12 g/d. Peripheral blood smear analysis revealed normocytic normochromic anemia with mild rouleaux formation. According to the serologic laboratory test, the anti-GBM antibody was positive (>200 IU/mL), and the anti-MPO antibody was positive (54.7 IU/mL; Table 1).

**Table 1:** Patient demographics and laboratory results on admission.

	Results		Results
Demographic variables		Urinalysis	
Height (cm)	169	Urine albumin	1+
Weight (kg)	79.7	Red blood cell (microscopy)	Many
Body mass index (kg/m^2^)	27.9	uPCR (g/gCr)	1.49
Laboratory variables		Serologic markers	
White blood cell (10^3^/µL)	6.9	CRP (mg/dL)	8.1
Hemoglobin (g/µL)	6.8	C3 (mg/dL)	108.9
Platelet (10^3^/µL)	263	C4 (mg/dL)	36.0
Blood urea nitrogen (mg/dL)	46	ASO (IU/mL)	40
Cr (mg/dL)	3.21	ANA	1:80
Estimated glomerular filtration rate (CKD-EPI) (mL/min/1.73 m^2^)	17.4	Anti-GBM antibody (U/mL)	>200
Sodium (mmol/L)	136	Anti-MPO antibody (U/mL)	54.7
Potassium (mmol/L)	4.6	Anti-PR3 antibody (U/mL)	2.1
Calcium (mg/dL)	7.5		
Corrected calcium (mg/dL)	8.9	HBsAg	Negative
Inorganic phosphate (mg/dL)	3.7	Anti-HCV	Negative
Total protein (g/dL)	7.3	Beta-2 microglobulin (mg/dL)	14.6
Albumin (g/dL)	2.3	Kappa-free light chain (mg/L)	504.7
Total bilirubin (mg/dL)	0.4	Lambda-free light chain (mg/L)	412.4
Lactic dehydrogenase (IU/L)	376	Free light chain ratio (%)	122.4

ANA = antinuclear antibody, anti-HCV = anti-hepatitis C virus antibody, ASO = antistreptolysin O, C3 = complement 3, C4 = complement 4, CKD-EPI = Chronic Kidney Disease Epidemiology Collaboration, Cr = creatinine, CRP = C-reactive protein, GBM = glomerular basement membrane, HBsAg = hepatitis B surface antigen, MPO = myeloperoxidase, PR3 = proteinase 3, uPCR = urine protein-to-creatinine ratio.

**Figure 1.**
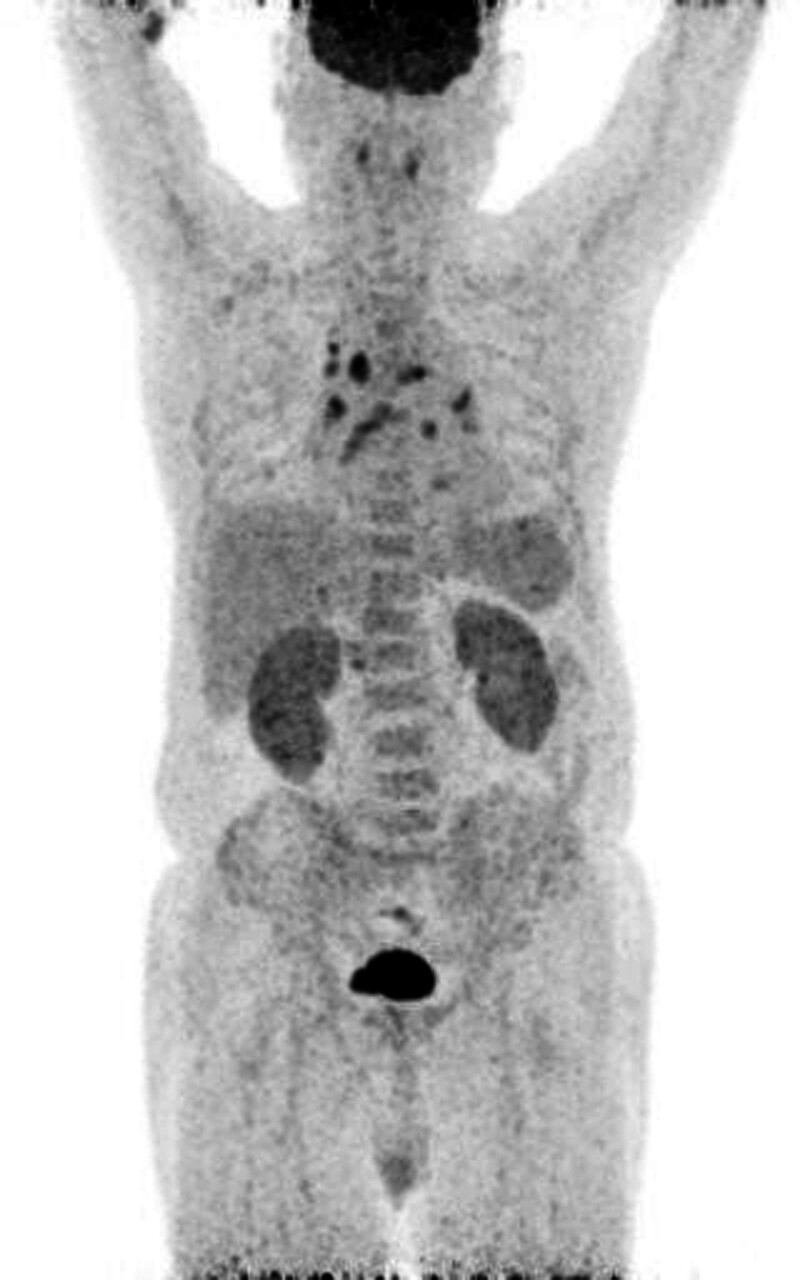
Fluorodeoxyglucose (FDG)-positron emission tomography scan of the torso. The presence of generalized heightened FDG uptake in both the bone marrow and spleen is noted, which prompted consideration of potential involvement of multiple myeloma. The mediastinal hypermetabolic lymph nodes 3A, 4R/L, 5, 6, 7, and 8R/L are considered reactive. FDG = fluorodeoxyglucose.

We observed a reversed albumin/globulin ratio, hypocalcemia, and anemia of chronic disease accompanied by mild rouleaux formation. There was no evidence of monoclonal gammopathy by protein electrophoresis or immunofixation. Additionally, the free light chain level was increased: free kappa chain level, 504.7 mg/L and free lambda chain level, 412.4 mg/L. Those ratio was 122.4%. Positron emission tomography-computed tomography was conducted to exclude other systemic malignancies representing generalized increased fluorodeoxyglucose uptake in the bone marrow and spleen, indicating myeloma involvement (Fig. 1).

To distinguish between the underlying cause of the patient’s disease and kidney dysfunction, we performed both kidney and bone marrow biopsies.

## 3. Pathological evaluation

**Figure 2.**
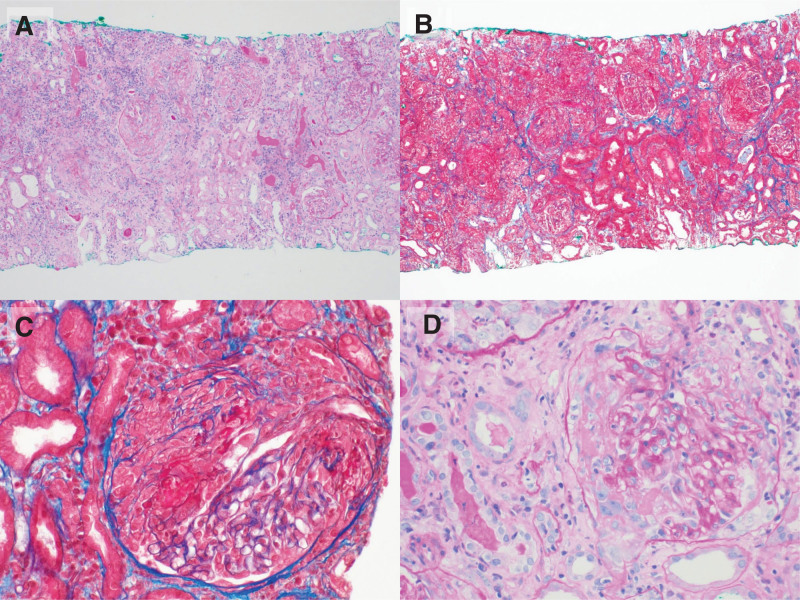
Light microscopy findings. Renal parenchyma showing 15 glomeruli with cellular and fibrocellular and fibrous crescents. The interstitium shows widespread inflammation and moderate tubular atrophy. There are also inflammatory cell infiltrates. The granulomatous lesion is not found. Panels A and B representing low-magnification (×100) observations, and panels C and D illustrating high-magnification (×400) observations. Specifically, panels A and C display results from Periodic acid-Schiff staining, while panels B and D present findings from Masson trichrome staining.

We obtained 3 cores of kidney tissue measuring 1 cm from ultrasound-guided kidney biopsy. Light microscopy showed no global or segmental sclerosis in the 15 glomeruli (Fig. 2). Cellular crescents were observed in 2 glomeruli, fibrocellular crescents in 10 glomeruli, and fibrous crescents in 2 glomeruli. Increased mesangial matrix and basement membrane thickening of the glomerulus and periglomerular fibrosis were not observed. The interstitium showed widespread inflammation, neutrophil infiltration, and moderate tubular atrophy. No granulomatous lesions or signs of myeloma or amyloidosis were observed. Immunofluorescence analysis supported the pauci-immune results, with a lack of immune complex deposition of immunoglobulin (Ig)-G, IgA, IgM, C3, C4, C1q, kappa, and lambda. Electron microscopy findings revealed no electron-dense deposits or GBM thickening, and there was no evidence of podocyte foot process effacement. Overall, the pathological findings indicated ANCA-associated crescentic glomerulonephritis, but not anti-GBM disease.

Bone marrow aspiration of the anterosuperior iliac crest was performed using an 11-gauge core needle. The touch prints of the aspirated bone marrow revealed 10% plasma cells with hypercellularity, while erythroid, myeloid, and megakaryocyte elements represented normal maturation. The bone marrow biopsy showed hypercellular bone marrow with stage 1 fibrosis. The specimen exhibited 60% cellularity, accompanied by increased plasma cell infiltration with 15% cellular components. Fluorescence in situ hybridization and karyotyping revealed no major genetic abnormalities associated with MM. Bony or extramedullary plasmacytoma was not detected; nonetheless, the observation of bone marrow plasma cells equal to or exceeding 10% aligned with the pathological features characteristic of MM.

## 4. Clinical course

On the basis of serological and pathological findings, we diagnosed the patient with anti-MPO-associated crescentic GN and MM without kidney involvement. During the third week of hospitalization, the patient exhibited persistent deterioration of kidney function, accompanied by alveolar hemorrhage and hemoptysis. Considering the possibility of AAV-related lesions, intravenous methylprednisolone (dose, 250 mg/d) for 3 days was administered as steroid pulse therapy. Although the respiratory lesions improved, kidney function did not recover, leading to the initiation of kidney replacement therapy through hemodialysis.

**Figure 3.**
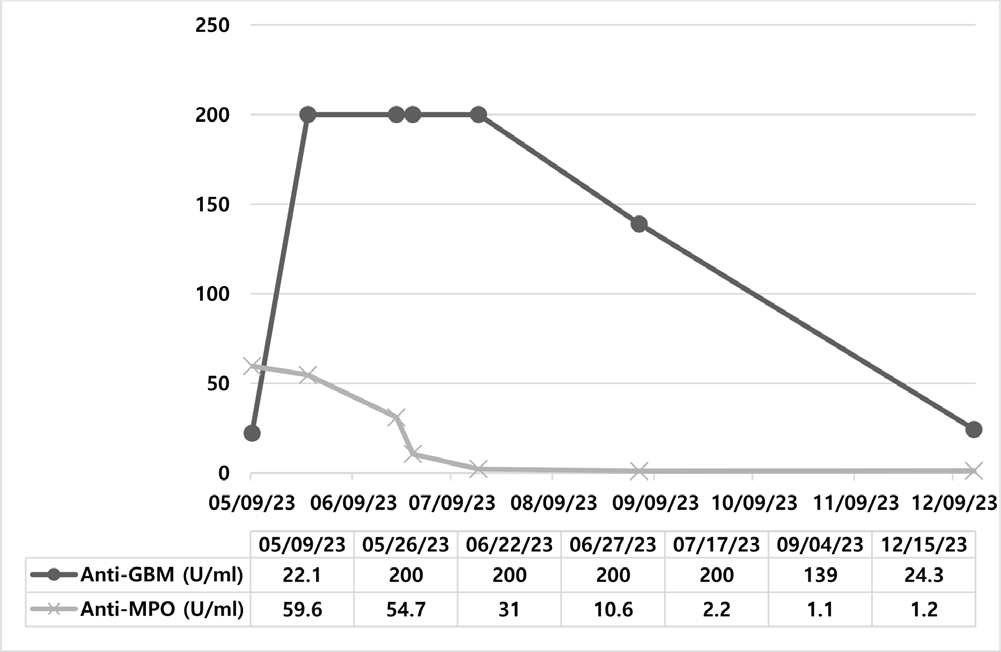
Serologic change in anti-GBM and anti-MPO antibodies. The levels of anti-GBM and anti-MPO antibodies decreased after the first diagnosis. Based on the latest test, conducted after 7 months thereafter, the anti-GBM antibody level was found to be 24.3 IU/mL, indicating a positive outcome. However, the anti-MPO antibody level was negative. GBM = glomerular basement membrane, MPO = myeloperoxidase.

A decline in the levels of anti-GBM and anti-MPO antibodies since the initial diagnosis was observed. According to the most recent test result, obtained on December 15, 2023, the anti-GBM antibody remained positive at 24.3 IU/mL, while the anti-MPO antibody was negative (Fig. 3). During the patient’s follow-up, we additionally investigated the levels of free light chains. Despite the patient being on maintenance dialysis, making it challenging to establish a correlation with kidney function, the ratio between kappa and lambda chains did not exhibit a significant change overall.

The consideration of therapeutic intervention for MM, distinct from that for AAV-associated crescentic GN, involves lenalidomide-based chemotherapy. Nevertheless, given the patient’s advanced age and an Eastern Cooperative Oncology Group stage of 3 to 4, which posed challenges in contemplating post-induction chemotherapy modalities such as autologous stem cell transplantation, the decision was made to abstain from chemotherapy and instead implement supportive care with maintenance dialysis.

## 5. Discussion

We described an extremely rare case involving the combination of 2 distinct disease entities: double-seropositive ANCA-associated GN and MM. On the basis of the clinical manifestations, the patient exhibited characteristics of RPGN along with severe hypoalbuminemia with subnephrotic range proteinuria. Moreover, laboratory findings indicated the possibility of MM, as evidenced by anemia, the reversed albumin-globulin ratio, and rouleaux formation observed on peripheral blood smear analysis. Finally, the specific disease was confirmed through kidney and bone marrow biopsies. Given the rarity of double-seropositive ANCA-associated GN, the coexistence of MM is uncommon. This case report not only underscores the importance of presenting an exceptionally rare disease but also highlights the indispensable role of a complex diagnostic process in attaining a definitive diagnosis.

The simultaneous detection of anti-GBM antibodies and ANCA in serological tests is uncommon, and its prevalence varies greatly, ranging from 7% to 41%.^[7]^ According to a European study of 646 individuals, 5.7% were positive according to both serological tests, with 64% exhibiting crescentic GN. Patients with double seropositivity shared characteristics of ANCA-associated vasculitis with older age, longer duration of symptoms, and features of anti-GBM disease with a severe phenotype of kidney dysfunction and alveolar hemorrhage.^[5]^ Interestingly, this case also shared these clinical characteristics with old age, kidney dysfunction representing RPGN features, and alveolar hemorrhage.

An association between ANCA and anti-GBM disease has been proposed on the basis of the sequential expression patterns of these 2 antibodies^[8–10]^; yet, the precise pathophysiological mechanism underlying this phenomenon remains unclear. However, it has been suggested that both antibodies share some key sequence homology and appear simultaneously, potentially leading to cross-reactive T-cell responses and mutual induction.^[11]^ Additionally, there is a possibility that patients with preexisting AAV may develop anti-GBM vasculitis due to epitope spreading.^[4]^ This conjecture provides evidence for the concurrent occurrence of these 2 conditions. There is an ongoing effort to understand the genetic connection between anti-GBM-associated disease and ANCA-associated vasculitis.^[12]^ It has been presented a potential avenue for the generation of anti-GBM antibodies by ANCA.^[12]^ Additionally, our patient exhibited a higher level of anti-GBM and anti-MPO antibodies but demonstrated pauci-immune pathological findings in the kidney tissue. This case demonstrates deviations in clinical characteristics compared with other cases.

The coexistence of seropositive vasculitis and malignancy is uncommon. According to Mahr et al,^[13]^ vasculitis therapy frequently results in procalcinogenic consequences that are mostly linked to the occurrence of cancer.Nevertheless, it is worth noting that in a limited number of cases, the presence of malignant diseases – particularly lymphoma and myeloma – preceded the occurrence of vasculitis and subsequently contributed to its development.^[14]^ Previous research has established the association between AAV and chronic lymphocytic leukemia. The study by Henriksen et al^[15]^ analyzed 6 patients exhibiting signs of chronic lymphocytic leukemia, RPGN, and pauci-immune glomerulonephritis based on kidney histology. Additionally, neoplastic lymphoid infiltration in kidney tissue was noted in some histologic stains. They hypothesized that this infiltration could be one of the mechanisms contributing to glomerular tissue inflammation.

The presence of monoclonal antibodies may correlate with the development of ANCA. Plasma cells play an important role in the development of AAV. The process of autoreactive B cell formation and their subsequent maturation into plasma cells that secrete ANCA is regarded as a crucial step in the development of AAV. Patients with AAV exhibit higher proportions of B lymphocytes and plasma cells than healthy controls.^[16,17]^ Other studies have shown that rheumatologic diseases like AAV affect the risk of MM or other lymphoproliferative disorders progressed from monoclonal gammopathy of undetermined significance (MGUS).^[18]^ In another cohort of 16 individual case studies, all patients were positive for anti-PR3 and anti-MPO antibodies and were diagnosed with MM. Despite the observation of ANCA-associated crescentic GN, none exhibited evidence of kidney involvement associated with MM.^[19]^ Cheta and Binder^[20]^ reported on 4 patients who exhibited monoclonal kappa/lambda immunoglobulin G positivity, suggesting a potential case of MM. Kidney histology analysis revealed pauci-immune glomerulonephritis in these patients. Notably, an 85-year-old female tested positive for both anti-MPO and anti-PR3 antibodies, while also displaying monoclonal kappa light chain and atypical casts on kidney histology. The study documented that 3 patients received VELCADE-based immunosuppressive therapy or plasma exchange and experienced partial improvement in kidney function. Unfortunately, 2 of these cases resulted in fatal pulmonary hemorrhage. The management of ANCA-associated vasculitis requires the implementation of glucocorticoid induction therapy including cyclophosphamide and rituximab, and subsequent maintenance therapy comprising rituximab or azathioprine in conjunction with low-dose glucocorticoids.^[21]^ However, there is an ongoing debate over the management and long-term prognosis of simultaneous seropositive vasculitis, and so far, there is a lack of comprehensive research on this subject. When confronted with the concurrent manifestation of multiple medical conditions, the selection of an appropriate treatment strategy necessitates consideration of not only established guidelines but also a comprehensive assessment of the patient’s overall clinical profile. In the context of this particular case, wherein double-seropositive ANCA-associated glomerulonephritis coexisted with MM, and given the advanced age and diminished performance status of the patient, the application of aggressive immunosuppressive therapy was limited. Consequently, a judicious approach was adopted, involving the implementation of low-dose steroid pulse therapy, followed by a conservative management strategy.

The renal prognosis of simultaneous seropositive vasculitis is comparable or somewhat superior to that of solitary anti-GBM vasculitis, but notably more severe than that observed in individuals with AAV.^[5]^ If the dominant phenotype is similar to anti-GBM disease, it appears with more severe renal symptoms and prognosis compared with those with severe renal failure in AAV.^[22]^ Although the presence of anti-GBM antibodies in patients with AAV does not appear to be linked to renal involvement, the findings suggest that these antibodies have prognostic importance in patients with AAV with renal involvement. In addition, ANCAs may serve as prognostic indicators in patients diagnosed with anti-GBM vasculitis.^[10]^

This study holds important value as a report on a remarkably rare medical condition. However, this study has several limitations. The inherent limitations of a case report design preclude an in-depth exploration of the pathophysiological aspects underlying disease onset. Additionally, the rarity of the condition necessitates an approach for diagnosis and treatment that has not been definitively established. Nevertheless, despite these challenges, the present case report contributes substantially to the accumulation of evidence regarding the characteristics of double-seropositive ANCA glomerulonephritis and the simultaneous occurrence of ANCA vasculitis and MM. Its importance lies in its potential to provide tangible knowledge on these rare coexisting entities, thus enhancing our understanding of their clinical manifestations and management.

## 6. Conclusions

This study described an exceptionally rare case involving the convergence of double-seropositive ANCA-associated glomerulonephritis and MM. The simultaneous detection of anti-GBM antibodies and ANCA, while uncommon, underscores the complexity of immune-mediated diseases and raises questions regarding potential shared pathophysiological mechanisms. Further research and case studies on this rare intersection of diseases are crucial to advance our knowledge and refine treatment strategies.

## Author contributions

**Data curation:** Hyeonjeong Lee.

**Investigation:** Hyeonjeong Lee, Jaeseok Yang, Jinykung Kwon, Jin Hyuk Paek, Seungyeup Han.

**Resources:** Mihwa Heo, Hyeongchan Shin, Misun Choe.

**Writing – original draft:** Hyeonjeong Lee.

**Writing – review & editing:** Yaerim Kim, Kyubok Jin.
